# *Notes from the Field:* Pediatric HIV Outbreak in Ratodero, Pakistan — April 2019–April 2020

**DOI:** 10.15585/mmwr.mm7042a5

**Published:** 2021-10-22

**Authors:** Elizabeth M. Rabold, Saqib Ali Shaikh, Karl Schenkel, Mirza Amir Baig, Rana Jawad Asghar, Ahmed Liban, Oliver Morgan, Hammad Ali

**Affiliations:** ^1^Epidemic Intelligence Service, CDC; ^2^Division of Global HIV & TB, Center for Global Health, CDC; ^3^Sindh AIDS Control Programme, Ministry of Health, Karachi, Pakistan; ^4^World Health Organization, Geneva, Switzerland; ^5^Pakistan Field Epidemiology and Laboratory Training Program, Islamabad, Pakistan; ^6^Global Health Strategists & Implementers, Islamabad, Pakistan; ^7^Division of Global Health Protection, Center for Global Health, CDC.

In April 2019, local media reports alerted the Sindh AIDS Control Program (SACP) in Pakistan of 14 children aged <10 years with new diagnoses of HIV infection in Ratodero, a rural subdistrict of Larkana District in Sindh province* (*1*). The report of pediatric cases of HIV infection in these children, whose parents all had received negative HIV test results, was concerning given the low number of children living with HIV in Pakistan (4,200 in a population of 79 million children) and the low (<0.1%) HIV prevalence estimate in the general population.^†^^,^^§^ Within 2 weeks, SACP, with assistance from the Pakistan Field Epidemiology and Laboratory Training Program (FELTP), established 18 health care and community testing sites throughout Larkana District to identify additional cases. Testing was limited to specimens from persons who visited these voluntary testing sites, regardless of symptoms, and did not include contact tracing of clients with positive test results for HIV infection or implementation of HIV testing at high-risk clinical entry points (e.g., emergency departments or infectious disease clinics).

By May 18, 2019, among 16,856 persons tested, health officials identified 571 (3.4%) new cases of HIV infection, 463 (81%) of which were in children and adolescents aged ≤15 years, including 355 (62%) aged ≤5 years.^¶^ In late May 2019, Pakistan’s Federal Health Ministry requested assistance from the World Health Organization (WHO). International partners including other United Nations agencies and CDC joined WHO to support SACP, FELTP, and other local partners in the outbreak investigation and response. Preliminary investigations (including patient interviews, site visits to clinics, hospitals, and blood banks, and review of surveillance data) identified unsafe injection practices at health care facilities, unsafe practices at blood banks, inadequate infection control measures, and improper management of medical waste as possible risk factors. The Expanded Programme on Immunization services in Pakistan, which includes immunizations against hepatitis B, tuberculosis, polio, and other childhood diseases, uses single-use, auto-disable syringes, and thus routine childhood vaccinations were not deemed to be associated with the outbreak. Because most of the children’s mothers were HIV-negative, mother-to-child transmission could not have occurred in most cases. The response team recommended improving infection prevention and control and blood safety, including educating health care workers about safe injection practices, convening task forces with critical stakeholders, and enforcing policy changes and regulations.

After the initial outbreak investigation, dedicated testing sites continued to identify more persons living with HIV. During April 2019–April 2020, a total of 1,353 persons (3.2%) received positive HIV test results in Ratodero. Approximately 75% of newly identified HIV infections occurred in children and adolescents aged <15 years, of which 633 (61%) were boys and 405 (39%) girls.** Consistent with preliminary outbreak investigation findings, a case-control study identified iatrogenic transmission as the predominant mode of HIV transmission, likely related to poor infection prevention and blood safety practices (*2*). Prevalence of hepatitis B surface antigen (18%) and hepatitis C antibodies (6.5%) among persons with a newly received diagnosis of HIV infection was higher than that in controls (5% and 1%, respectively) and that of the pediatric population in the same province (1.8% and 1.6%, respectively) (*3*,*4*). A pending phylogenetic analysis might provide additional information about potential routes of HIV transmission.

Iatrogenic transmission of HIV has been associated with at least four other HIV outbreaks in Pakistan during the past 20 years (*5*); the high prevalence of hepatitis B and C in the country raises concern for iatrogenic transmission of other bloodborne pathogens. Improvements at the local and national levels in health care practices, community education, and health care provider training with an emphasis on infection prevention and control measures, could help prevent future outbreaks of HIV and other bloodborne infections in Pakistan.

## Footnotes

*
https://www.nytimes.com/2021/03/31/magazine/pakistan-hiv.html

†
https://aidsinfo.unaids.org/

§
https://dhsprogram.com/pubs/pdf/FR354/FR354.pdf

¶
https://www.nih.org.pk/field-epidemiology-laboratory-training-program-feltp-2/

** https://nacp.gov.pk. Accessed April 30, 2020.
